# Impact of Patient Demographics and Cardiovascular Risk Factors on Percutaneous Coronary Intervention Outcomes

**DOI:** 10.7759/cureus.106076

**Published:** 2026-03-29

**Authors:** Syed Muhammad Nayab Ali, Zahid Ullah, Saddam Hussain, Sher Ali Khan, Aimal K Afridi, Syed Misbah Uddin

**Affiliations:** 1 Department of Cardiology, Lady Reading Hospital, Peshawar, PAK; 2 Department of Cardiology, Khyber Teaching Hospital Medical Teaching Institution (MTI), Peshawar, PAK; 3 Department of Medicine, Lady Reading Hospital, Peshawar, PAK

**Keywords:** cardiovascular risk factors, coronary artery disease, major adverse cardiovascular events, patient demographics, percutaneous coronary intervention

## Abstract

Background: Percutaneous coronary intervention (PCI) is extensively employed for the therapy of coronary artery disease; nonetheless, patient demographics and cardiovascular risk factors continue to impact procedural and clinical results

Objective: To evaluate the association between patient demographics and cardiovascular risk factors and in-hospital clinical outcomes, including major adverse cardiovascular events (MACE), stent thrombosis, and mortality, among patients undergoing PCI.

Methodology: This hospital-based observational study was conducted over a one-year period from July 2023 to June 2024. A total of 270 adult patients undergoing elective or emergency PCI were enrolled using a convenience sampling technique. Data on demographic characteristics, cardiovascular risk factors, procedural details, and in-hospital outcomes were collected using a structured proforma designed by hospital staff and research investigators. Statistical analysis was performed using SPSS version 26 (IBM Corp., Armonk, New York, USA), including chi-square tests, independent-samples t-tests, and multivariate logistic regression to identify independent predictors of adverse outcomes.

Results: Of the 270 patients, 185 (68.52%) were male and 93 (34.44%) were aged ≥60 years. Hypertension was present in 158 (58.52%) patients, and diabetes mellitus in 120 (44.44%) patients. Procedural success was achieved in 250 (92.59%) cases, while in-hospital major adverse cardiovascular events (MACE) occurred in 20 (7.41%), stent thrombosis in 8 (2.96%), and mortality in 5 (1.85%) patients. Patients who developed MACE were significantly older than those with procedural success (61.7 ± 9.5 vs. 55.3 ± 10.2 years; p = 0.034). Multivariate analysis identified age ≥60 years (adjusted OR: 2.10; p = 0.035) and diabetes mellitus (adjusted OR: 3.25; p = 0.003) as independent predictors of adverse PCI outcomes.

Conclusion: Advanced age and diabetes mellitus were significant determinants of adverse in-hospital outcomes following PCI despite high overall procedural success rates.

## Introduction

A key component of the treatment of coronary artery disease (CAD), percutaneous coronary intervention (PCI), provides patients with both acute coronary syndromes and stable ischemic heart disease with efficient revascularization [1]. Advances in interventional procedures, stent technology, and concomitant medication have considerably improved procedural success rates and short-term outcomes [2]. Variability in clinical outcomes after PCI, however, continues to be a persistent worry despite these developments, indicating that patient-related variables continue to be critical in predicting prognosis [3].

Patient demographics, such as age and sex, have been demonstrated to impact cardiovascular disease presentation, treatment options, and post-procedural outcomes [4]. Older patients generally present with complicated coronary architecture and many comorbidities, which may increase the risk of adverse events during PCI [5]. Disparities in procedural results and recovery patterns may also result from sex-based variations in cardiovascular pathophysiology, artery size, and responsiveness to medication [6]. Optimizing interventional techniques and improving patient selection requires an understanding of these demographic factors [7].

In addition to demographic considerations, classic cardiovascular risk factors--including hypertension, diabetes mellitus, dyslipidemia, smoking, and obesity--play a crucial role in the development and progression of atherosclerosis [8]. These risk factors may impact endothelial function, inflammatory response, and vascular repair after intervention, in addition to increasing the severity and degree of coronary artery disease [9]. Patients with several co-existing risk factors frequently demonstrate more widespread disease and may face greater rates of residual stenosis, stent thrombosis, or other post-PCI problems [10].

The relationship between patient demographics and cardiovascular risk profiles is complicated and may jointly impact both immediate procedural success and short-term clinical outcomes after PCI [11]. In different populations, especially in low- and middle-income nations, changes in demographic features and risk factor load may further influence outcomes, underlining the need for population-specific evidence [12]. A detailed study of these parameters may give useful insights into result variability and promote more customized approaches to interventional cardiology. The objective of this study was to evaluate the impact of patient demographics and cardiovascular risk factors on clinical outcomes following PCI.

## Materials and methods

Study design and setting

The Lady Reading Hospital, Peshawar, was the site of this hospital-based observational research. The research ran from July 2023 to June 2024 for a total of one year. In order to evaluate procedural and early clinical outcomes, patients receiving PCI during the research period were recruited and monitored throughout their hospital stay.

Inclusion and exclusion criteria

Patients of either sex aged 18 years or older who had PCI for the therapy of coronary artery disease were included in the research. Both elective and emergency PCI patients were deemed eligible. To guarantee accurate data analysis, patients with comprehensive demographic data and established cardiovascular risk profiles were included. Patients with inadequate medical records, concurrent cardiac surgeries during the same hospitalization, or a history of past coronary artery bypass graft surgery were excluded. Additionally, patients with serious non-cardiac comorbid illnesses that might independently impact short-term results or who refused to participate were omitted.

Sample size

A total of 246 patients were initially calculated for enrollment based on sample size estimation. To account for potential missing or incomplete data, the sample size was increased by 10%, resulting in a final cohort of 270 patients, representing all eligible PCI cases during the study period with complete demographic, clinical, and procedural data.

Data collection

Data were gathered using a standardized proforma developed specifically for this study by hospital personnel and research investigators. Demographic variables included age and sex, while patient history, clinical assessment, and medical records were used to document cardiovascular risk factors such as obesity, diabetes mellitus, dyslipidemia, smoking status, and hypertension. Procedural information and PCI results were obtained from catheterization laboratory reports and hospital records. To ensure accuracy and consistency, all data collection was performed by trained investigators and hospital personnel.

Primary outcomes were procedural success, in-hospital major adverse cardiovascular events (MACE), stent thrombosis, and death. MACE was defined as a composite of in-hospital mortality, myocardial infarction, and target vessel revascularization. Secondary outcomes, measured during the in-hospital stay, included post-PCI complications, such as residual stenosis, bleeding episodes, and length of hospital stay. Residual stenosis was defined as >20% diameter stenosis remaining immediately after PCI, assessed angiographically at the end of the procedure. This should not be interpreted as late restenosis, which typically develops months after stent implantation.

Operational definitions

MACE: Composite of in-hospital mortality, myocardial infarction, and target vessel revascularization.

Procedural success: Successful completion of PCI without immediate major complications.

Stent thrombosis: Occurrence of thrombotic occlusion in the treated stent during hospitalization.

Residual stenosis: >20% diameter stenosis remaining at the end of the PCI procedure, assessed angiographically. Not to be interpreted as late restenosis.

Bleeding events: Any clinically significant bleeding occurring during hospitalization (e.g., requiring transfusion or intervention).

Length of hospital stay: Duration of inpatient stay following PCI, measured in days.

Age: Reported as both a continuous variable (years) and a categorical variable (≥60 years).

Obesity: Body mass index (BMI) ≥30 kg/m².

Cardiovascular risk factors: Presence of hypertension, diabetes mellitus, dyslipidemia, and smoking, defined according to standard clinical criteria.

Lesion complexity: Complex lesions identified during angiography.

Access site: Radial or femoral arterial access for PCI.

Stent type: Drug-eluting stent or bare-metal stent.

Number of treated vessels: Single-vessel vs. multi-vessel PCI.

Clinical presentation: ST-elevation myocardial infarction (STEMI), non-ST-elevation myocardial infarction (NSTEMI), or stable CAD.

Statistical analysis

Version 26 of the Statistical Package for Social Sciences (SPSS) (IBM Corp., Armonk, New York, USA) was used to analyze the data. Categorical data were shown as frequencies and percentages, while continuous variables were given as mean ± standard deviation. Using appropriate statistical methods, including the independent-samples t-test when appropriate and the chi-square test for categorical data, associations between patient demographics, cardiovascular risk factors, and PCI outcomes were evaluated. Age was analyzed both as a continuous variable to compare mean differences between groups and as a categorical variable (≥60 years) to assess clinically meaningful thresholds in categorical and regression analyses. To assess the independent influence of patient demographics and cardiovascular risk variables on PCI results, multivariate logistic regression analysis was used. A p-value of <0.05 was judged statistically significant.

Ethical approval

Ethical approval for the study was obtained from the Institutional Review Board of Lady Reading Hospital, Peshawar. Written informed consent was obtained from all participants prior to enrollment. Confidentiality of patient information was strictly maintained, and the study was conducted in accordance with the principles of the Declaration of Helsinki.

## Results

Table 1 summarizes the baseline characteristics, cardiovascular risk factors, and PCI outcomes of the study population (n = 270). Most patients were aged 40-59 years (48.89%), followed by those ≥60 years (34.44%), and males constituted 68.52% of the cohort. Hypertension (58.52%) and diabetes mellitus (44.44%) were the most prevalent risk factors, while 28.89% of patients had three or more cardiovascular risk factors. Procedural success was achieved in 92.59% of cases, with in-hospital MACE occurring in 7.41%, stent thrombosis in 2.96%, and mortality in 1.85%. Among secondary outcomes, prolonged hospital stay (>5 days) was observed in 12.96%, bleeding events in 5.56%, and residual stenosis in 4.44% of patients.

**Table 1:** Baseline characteristics, cardiovascular risk factors, and in-hospital PCI outcomes (n = 270). PCI: percutaneous coronary intervention. MACE: major adverse cardiovascular events, defined as a composite of in-hospital mortality, myocardial infarction, and target vessel revascularization. Stent thrombosis was not included in the MACE composite and is reported separately.

Variable	Category	n (%)
Age (years)	<40	45 (16.67)
	40–59	132 (48.89)
	≥60	93 (34.44)
Sex	Male	185 (68.52)
	Female	85 (31.48)
Hypertension	Yes	158 (58.52)
	No	112 (41.48)
Diabetes mellitus	Yes	120 (44.44)
	No	150 (55.56)
Dyslipidemia	Yes	95 (35.19)
	No	175 (64.81)
Smoking	Yes	102 (37.78)
	No	168 (62.22)
Obesity (BMI ≥30 kg/m²)	Yes	80 (29.63)
	No	190 (70.37)
≥3 Risk factors	Yes	78 (28.89)
	No	192 (71.11)
Primary PCI outcomes	Success	250 (92.59)
	MACE (composite)	20 (7.41)
	In-hospital mortality	5 (1.85)
Secondary (non-MACE) outcomes	Stent thrombosis	8 (2.96)
	Residual stenosis	12 (4.44)
	Bleeding events	15 (5.56)
	Prolonged hospital stay (>5 days)	35 (12.96)

Table 2 presents comparisons of continuous variables by PCI outcomes using independent-samples t-tests. Patients who developed MACE were significantly older than those with procedural success (61.7 ± 9.5 vs. 55.3 ± 10.2 years; p = 0.034). Additionally, patients experiencing post-PCI complications had a significantly longer hospital stay compared to those without complications (6.0 ± 1.5 vs. 4.2 ± 1.1 days; p = 0.021).

**Table 2:** Comparison of continuous variables according to MACE and post-PCI complications (independent sample t-test). MACE: major adverse cardiovascular events; PCI: percutaneous coronary intervention. Independent sample t-test was used for comparison. A p-value <0.05 was considered statistically significant.

Variable	Outcome group	n	Mean ± SD	Mean difference	t-value	p-value
Age (years)	No MACE (procedural success)	250	55.3 ± 10.2	6.4	-2.13	0.034
	MACE	20	61.7 ± 9.5			
Length of hospital stay (days)	No post-PCI complications	235	4.2 ± 1.1	1.8	-2.32	0.021
	With post-PCI complications	35	6.0 ± 1.5			

Table 3 shows the association between patient demographics, cardiovascular risk factors, and primary PCI outcomes. A significantly higher proportion of patients aged ≥60 years experienced MACE compared to those <60 years (13.98% vs. 3.41%; p = 0.045). Diabetes mellitus was also significantly associated with adverse outcomes, with MACE occurring in 12.50% of diabetic patients versus 3.33% of non-diabetic patients (p = 0.030). No statistically significant associations were observed for sex (p = 0.120) or hypertension (p = 0.055).

**Table 3:** Association of patient demographics and cardiovascular risk factors with MACE (univariate analysis). MACE: major adverse cardiovascular events. Odds ratios (OR) are unadjusted. Odds ratios (OR) with 95% confidence intervals were calculated using univariate analysis. For each variable, one category was designated as the reference group (OR = 1.00) against which other categories were compared. Chi-square test was used for comparison. p < 0.05 is considered statistically significant.

Factor	Category	No MACE n (%)	MACE n (%)	OR (95% CI)	χ²	p-value
Age (years)	<60 (n=177)	171 (96.61%)	6 (3.39%)	Reference	4.02	0.045
	≥60 (n=93)	79 (84.95%)	14 (15.05%)	3.10 (1.15–8.32)		
Sex	Male (n=185)	170 (91.89%)	15 (8.11%)	1.45 (0.50–4.20)	2.42	0.120
	Female (n=85)	80 (94.12%)	5 (5.88%)	Reference		
Diabetes mellitus	Yes (n=120)	105 (87.50%)	15 (12.50%)	4.10 (1.45–11.50)	4.70	0.030
	No (n=150)	145 (96.67%)	5 (3.33%)	Reference		
Hypertension	Yes (n=158)	145 (91.77%)	13 (8.23%)	1.35 (0.52–3.50)	3.68	0.055
	No (n=112)	105 (93.75%)	7 (6.25%)	Reference		
Dyslipidemia	Yes (n=95)	85 (89.47%)	10 (10.53%)	1.75 (0.70–4.35)	1.85	0.170
	No (n=175)	165 (94.29%)	10 (5.71%)	Reference		
Smoking	Yes (n=102)	90 (88.24%)	12 (11.76%)	2.10 (0.85–5.15)	2.95	0.086
	No (n=168)	160 (95.24%)	8 (4.76%)	Reference		
Obesity (BMI ≥30 kg/m²)	Yes (n=80)	72 (90.00%)	8 (10.00%)	1.55 (0.60–4.00)	1.20	0.270
	No (n=190)	178 (93.68%)	12 (6.32%)	Reference		
≥3 Risk factors	Yes (n=78)	68 (87.18%)	10 (12.82%)	2.25 (0.90–5.60)	3.10	0.078
	No (n=192)	182 (94.79%)	10 (5.21%)	Reference		

Table 4 illustrates the results of multivariate logistic regression analysis identifying independent predictors of adverse PCI outcomes. Age ≥60 years was associated with a twofold increased risk of adverse outcomes (adjusted OR: 2.10; 95% CI: 1.05-4.21; p = 0.035), while diabetes mellitus emerged as a strong independent predictor, increasing the odds by more than three times (adjusted OR: 3.25; 95% CI: 1.50-7.05; p = 0.003). Other variables, including sex, hypertension, smoking, dyslipidemia, and obesity, were not independently associated with PCI outcomes.

**Table 4:** Multivariate logistic regression analysis for predictors of MACE. MACE: major adverse cardiovascular events; OR: odds ratio; CI: confidence interval. Adjusted odds ratios were derived using multivariate logistic regression analysis. The reference category for each variable was assigned an OR of 1.00. Only variables with complete data were included. Model interpretation should be cautious due to the limited number of outcome events.

Predictor	Category	Adjusted OR (95% CI)	p-value
Age (years)	<60	1.00 (Reference)	0.035
	≥60	2.10 (1.05–4.21)	
Sex	Female	1.00 (Reference)	0.65
	Male	1.18 (0.58–2.38)	
Diabetes mellitus	No	1.00 (Reference)	0.003
	Yes	3.25 (1.50–7.05)	
Hypertension	No	1.00 (Reference)	0.28
	Yes	1.45 (0.73–2.88)	
Smoking	No	1.00 (Reference)	0.08
	Yes	1.90 (0.92–3.90)	
Dyslipidemia	No	1.00 (Reference)	0.42
	Yes	1.35 (0.65–2.81)	
Obesity (BMI ≥30 kg/m²)	No	1.00 (Reference)	0.80
	Yes	1.10 (0.50–2.43)	

**Figure 1:**
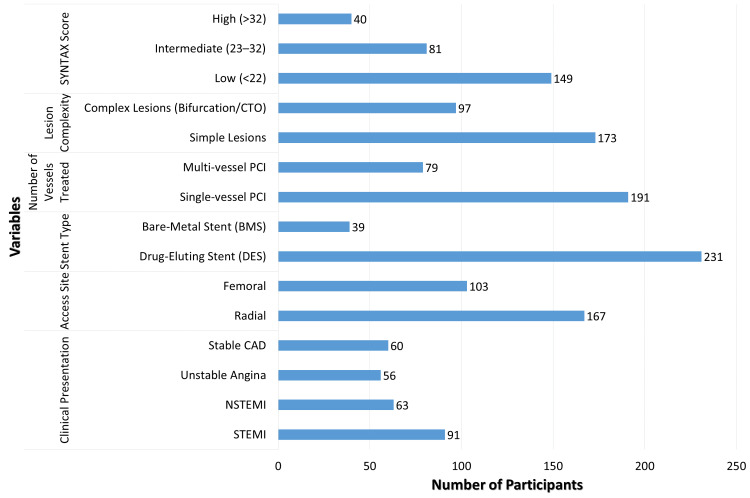
Procedural and angiographic characteristics of PCI (n = 270). PCI: percutaneous coronary intervention; CAD: coronary artery disease; STEMI: ST-elevation myocardial infarction; NSTEMI: non-ST-elevation myocardial infarction.

The procedural and angiographic characteristics of the research cohort are encapsulated in Figure 1. The majority of patients had acute coronary syndromes, comprising 91 individuals (33.70%) with ST-elevation myocardial infarction and 63 individuals (23.33%) with NSTEMI. Radial access was utilized in 167 individuals (61.85%), whilst femoral access was employed in 103 patients (38.15%). Drug-eluting stents were utilized in 231 patients (85.56%), whereas the remainder received bare-metal stents. The majority of patients received single-vessel PCI (191 patients, 70.74%), whereas multi-vessel PCI was conducted in 79 patients (29.26%). Complex lesions were detected in 97 individuals (35.93%). Anatomical risk assessment utilizing the SYNTAX score [13] revealed low scores in 149 individuals (55.19%), moderate scores in 81 patients (30.00%), and high scores in 40 patients (14.81%).

## Discussion

The distribution of important demographic traits and cardiovascular risk factors in this research of 270 PCI patients was noteworthy: men made up 68.52% of the cohort, and the majority were middle-aged (48.89% aged 40-59). Hypertension (58.52%) and diabetes mellitus (44.44%) were the most frequent risk variables, showing a significant burden of comorbidity. These prevalence estimates are congruent with data from observational studies in other countries; for example, a retrospective review of 224 PCI patients in Ethiopia found a similar preponderance of male sex and high hypertension prevalence (~56.7%) among patients having PCI [14]. These similarities imply that cardiovascular risk profiles in PCI populations may be generally comparable across varied geographic settings, underscoring the necessity of demographic and clinical risk characterization before intervention.

Age revealed as a key determinant in unfavorable outcomes in our population. Patients who had major adverse cardiovascular events (MACE) were substantially older (61.7 ± 9.5 years) compared with those with procedural success (55.3 ± 10.2 years; p = 0.034). This is consistent with a larger body of research showing that older patients have poorer clinical outcomes after PCI. A major pooled study of PCI studies found that women were older on average and reported greater rates of unadjusted unfavorable outcomes than men at five years [15]. Although our analysis did not show female sex to be an independent predictor, the age-related increase in poor outcomes reiterates that older patients may have more complicated illnesses and a comorbidity load, compromising PCI results.

Diabetes mellitus was independently associated with a higher risk of unfavorable PCI outcomes in our multivariate model (adjusted OR: 3.25; p = 0.003). These results are reinforced by meta-analytic evidence that diabetes is a substantial risk factor for in-stent residual stenosis and MACE following PCI, with many studies indicating increased event rates among diabetic patients [16]. For example, meta-analysis results identified diabetes as a factor raising in-stent residual stenosis risk as well as greater pooled chances for MACE, underlining that metabolic dysregulation continues to have a critical effect on PCI prognosis.

Although hypertension was frequent (58.52%) in our sample, it did not achieve statistical significance in multivariate analysis. Systematic studies have indicated that hypertension is commonly related to higher MACE risk; however, effects may diminish when corrected for co-existing risk factors [17]. Our results replicate such subtle connections, indicating that hypertension’s impact on outcomes may be interdependent with other variables like age and diabetes.

Stent thrombosis (2.96%) and in-hospital mortality (1.85%) were found to be comparatively low rates in our research. These result rates are similar to current PCI registries, where improved stent platforms and tailored medication have minimized acute complications. For instance, national registry data reveal composite short-term adverse event rates in the low single digits across large, contemporaneous cohorts [18]. Such consistency shows that procedure modification and risk stratification may help preserve good immediate results despite considerable baseline risk factor prevalence.

Length of hospital stay was considerably higher among patients with complications (6.0 ± 1.5 vs. 4.2 ± 1.1 days; p = 0.021), showing that unfavorable events delay recovery and resource consumption. Similar findings have been described in other clinical situations where complications, including MACE and hemorrhage, increase inpatient duration and healthcare burden [19]. These trends underline that beyond mortality and event rates, continuous measurements of clinical course are critical in understanding the entire effect of patient-level risk on PCI outcomes. These findings are particularly relevant in low- and middle-income countries, where delayed presentation, limited access to specialized care, and a higher burden of metabolic risk factors such as diabetes may adversely influence PCI outcomes.

Our findings regarding the prevalence of key risk factors and their impact on PCI outcomes are consistent with prior international and regional studies. Similar male predominance and high rates of hypertension and diabetes have been reported in observational cohorts from Ethiopia, India, and other low- and middle-income countries, highlighting a broadly comparable cardiovascular risk profile across diverse populations [14,20,21,22]. The observed association of advanced age and diabetes with increased risk of MACE aligns with large multicenter registries and meta-analyses, which consistently identify these factors as independent predictors of in-hospital and short-term adverse outcomes following PCI [15,16]. While hypertension was common in our cohort, its lack of independent significance mirrors findings from prior studies suggesting that its effect on outcomes may be confounded by co-existing conditions such as diabetes and older age [17,23]. Moreover, the low rates of stent thrombosis and in-hospital mortality in our study are comparable to those reported in contemporary PCI registries employing modern drug-eluting stents and optimized pharmacotherapy, reinforcing that procedural success can be achieved even in high-risk populations [18,24,25]. These comparisons support the external validity of our results and underscore the importance of pre-procedural risk stratification, targeted management of metabolic comorbidities, and careful post-PCI monitoring to optimize patient outcomes.

Study strengths and limitations

This study has several strengths. It was a hospital-based observational study conducted in a high-volume tertiary care center, ensuring access to comprehensive procedural and outcome data. The final sample included 270 patients, with a structured proforma used by trained hospital personnel to systematically collect demographic characteristics, cardiovascular risk factors, and in-hospital PCI outcomes, including procedural success, MACE, stent thrombosis, and mortality. The use of both univariate and multivariate statistical analyses allowed identification of independent predictors of adverse PCI outcomes, specifically age ≥60 years and diabetes mellitus.

However, certain limitations should be acknowledged. Although all eligible PCI patients presenting during the study period were approached for enrollment, the study employed convenience sampling, which may introduce selection bias. Being a single-center study, the findings may not be fully generalizable to other healthcare settings or populations. Outcomes were assessed only during the in-hospital period, precluding evaluation of long-term events such as late restenosis, target vessel revascularization, or post-discharge MACE. Residual confounding from unmeasured procedural variables, including lesion complexity, stent type, number of vessels treated, and access site, cannot be entirely excluded despite adjustment for key clinical and demographic factors reported in our tables and analyses.

## Conclusions

This study demonstrates that certain cardiovascular risk factors and patient demographics are associated with in-hospital outcomes after PCI. Specifically, advanced age and diabetes mellitus emerged as significant predictors of major adverse cardiovascular events, whereas sex, hypertension, and obesity were not independently associated with in-hospital complications. These findings highlight the importance of thorough pre-procedural risk assessment and careful management of metabolic comorbidities to optimize PCI outcomes. While procedural success rates were high, reflecting the effectiveness of contemporary PCI techniques, the associations observed should be interpreted with caution due to the observational, single-center study design and limited follow-up. Further multicenter and prospective studies are warranted to confirm these relationships and evaluate longer-term outcomes, including late restenosis and post-discharge adverse events.
